# Transplantation of Autologous Bone Marrow Mesenchymal Stem Cells with Platelet-Rich Plasma Accelerate Distraction Osteogenesis in A Canine Model

**DOI:** 10.22074/cellj.2016.3724

**Published:** 2015-07-11

**Authors:** Mohammad Mehdi Dehghan, Mohamadreza Baghaban Eslaminejad, Nader Motallebizadeh, Javad Ashrafi Halan, Leila Tagiyar, Sarang Soroori, Agbibi Nikmahzar, Mirsepehr Pedram, Abdolhossein Shahverdi, Hossein Kazemi Mehrjerdi, Sadra Izadi

**Affiliations:** 1 Department of Clinical Sciences, Faculty of Veterinary Medicine, University of Tehran, Tehran, Iran; 2 Department of Stem Cells and Developmental Biology at Cell Sciences Research Center, Royan Institute for Stem Cell Biology and Technology, ACECR, Tehran, Iran; 3 Limb Lengthening of Iran, Tehran, Iran; 4 Department of Clinical Sciences, Faculty of Veterinary Medicine, Tabriz University, Tabriz, Iran; 5 Department of Clinical Sciences, Faculty of Veterinary Medicine, Ferdowsi University of Mashhad, Mashhad, Iran

**Keywords:** Distraction Osteogenesis, Bone Lengthening, Mesenchymal Stem Cells, Autologous Transplantation, Platelet-Rich Plasma

## Abstract

**Objective:**

Distraction osteogenesis (DO) is a surgical procedure used to generate large volumes of new bone for limb lengthening.

**Materials and Methods:**

In this animal experimental study, a 30% lengthening of the left tibia (mean distraction distance: 60.8 mm) was performed in ten adult male dogs by callus distraction after osteotomy and application of an Ilizarov fixator. Distraction was started on postoperative day seven with a distraction rate of 0.5 mm twice per day and carried out at a rate of 1.5 mm per day until the end of the study. Autologous bone marrow mesenchymal stem cells (BM-MSCs) and platelet-rich plasma (PRP) as the treatment group (n=5) or PRP alone (control group, n=5) were injected into the distracted callus at the middle and end of the distraction period. At the end of the consolidation period, the dogs were sacrificed after which computerized tomography (CT) and histomorphometric evaluations were performed.

**Results:**

Radiographic evaluationsrevealed that the amount and quality of callus formations were significantly higher in the treatment group (P<0.05). As measured by CT scan, the healing parametersin dogs of the treatment group were significantly greater (P<0.05). New bone formation in the treatment group was significantly higher (P<0.05).

**Conclusion:**

The present study showed that the transplantation of BM-MSCs positively affects early bony consolidation in DO. The use of MSCs might allow a shortened period of consolidation and therefore permit earlier device removal.

## Introduction

Distraction osteogenesis (DO) is a surgical procedure by which controlled displacement of bone fragments is used to induce the generation of large volumes of new bone. This technique has been successfully used for limb lengthening (1). In this biomechanical process for skeletal lengthening, the formation of new bone along the distraction stress line is stimulated by exerting strain on both ends of the bony segments (2). This technique was first described by Codivilla (3), but did not gain wide acceptance until the physiological and mechanical aspects required for the successful regeneration of new bone were identified by Ilizarov (4). This technique is currently a standard and acceptable method for bone lengthening. DO is usually separated into three phases: i. the latency phase, immediately following the osteotomy and before distraction, ii. The distraction phase, characterized by the active distraction of the segments for a certain time at specific rates and frequencies and iii. the consolidation phase, characterized by bony union and mineralization. The duration of the consolidation depends on the distraction site, the status of vascularization, and the age of the patient (5). Although DO can produce reliable callus formation without grafting, many surgeons prefer to graft autologous can cellous bone (the current gold standard) for a better outcome (6). DO is used for complications of fractures such as nonunions, chronic osteomyelitis, shortened extremities, joint contractures, deformities and bone loss due to trauma, infections, or tumor resections (1). It should be mentioned that the use of this method has some limitations, such as the need for expensive devices (4), long-term treatment resulting in a high rate of complications such as infection, pin loosening, fracture, adjacent joint contractures, soft tissue swelling, and pain (5). Many attempts have been made to promote bone formation by increasing the distraction rate and shortening the consolidation phase, including electrical stimulation (7), hyperbaric oxygen exposure (8), low-intensity pulsed ultrasound stimulation (9), controlled mechanical stimulation (10), the injection of cytokines into the distracted callus (11), the transfusion of marrow cells or cultured periosteal cells (12), and other medical treatments. Among these strategies, tissue engineering in combination with osteogenic cells such as stem cells has been accepted as a possible alternative to accelerate bone regeneration (12). During DO, a series of cellular processes and complex molecular events induce the differentiation of mesenchymal stem cells (MSCs) into boneforming cells and, eventually, the formation of a distraction callus (13).

MSCs are adult stem cells that can be derived from many tissues, in particular bone marrow (BM) tissue. By far multiple studies have confirmed the great potential of MSCs in promoting regeneration of bone defects both in animal models and humans (14, 15). Scientists believe that MSCs can help regeneration by two ways: differentiation into tissue cells in order to restore lost morphology as well as function and secretion of a wide spectrum of bioactive factors that help to create a repair environment by possessing immunoregulatory function, anti-apoptotic effects, and the stimulation of endothelial progenitor cell proliferation (16). On the other hand, numbers of research works have reported that platelet-rich plasma (PRP) canenhancethehealing process in bone injuries (17, 18). The bone regenerative effects of PRP have also been reported at early phases of DO (19, 20). Some research has indicated that the addition of MSC into a PRP scaffold would be beneficial for increased new bone formation, mineralization, and mechanical property (21).

The present study evaluated the possibility of an increased distraction rate and decreased consolidation phase by using MSCs as an exogenous source of osteogenic progenitor cells for DO. This experimental study investigated whether the transplantation of MSCs and PRP could accelerate the events of DO or if these cells could influence different aspects of DO.

## Materials and Methods

### Animals

We used 10 healthy adult male mongrel dogs that ranged between 2 to 4 years of age and weighed 15 to 25 kg in this experimental study. The dogs were randomly divided into two groups: treatment (n=5) and control (n=5). All experiments were performed by authorization of the Animal Ethics Committee of the Royan Institute (Tehran, Iran).

### Mesenchymal stem cells isolation

Canine MSCs were isolated according to the method of Kadiyala et al. (22) with some modification from the dogs’ BM three weeks before the first transplantation of stem cells. Briefly, the nucleated cell fraction of the marrow was enriched by gradient centrifugation and cultured in 150-cm^2^ flasks at 5.0×10^4^ cells/ml in 15.0 ml low-glucose Dulbecco modified eagle medium (DMEM, Gibco, Germany) that contained 15% fetal calf serum (FCS, Gibco, Germany), 100 U/ml penicillin G and 100 U/ml streptomycin (Gibco, Germany). The cells were incubated at 37˚C in a humidified 5% CO_2_ atmosphere. On day seven, the non-adherent cells were removed along with the culture media. The cultures were fed twice a week, passaged on days 17-21 by lifting the cells with 0.05% Trypsin-0.53 mM Ethylenediaminetetraacetic acid (EDTA) (Gibco, Germany) exposure for five minutes and split in a 1:3 ratio into new 150-cm^2^ culture flasks.

### Differentiation potential

To evaluate the nature of the MSCs, the isolated cells were induced to differentiate into osteogenic, chondrogenic, and adipogenic cell lineages.

For osteogenic differentiation, confluent passage-3 cells were cultured in DMEM medium supplemented with 50 mg/ml L-ascorbic 2-phosphate (Sigma, USA), 10 nM dexamethasone and 10 mM β-glycerophosphate (Sigma, USA) for 3 weeks. For adipogenesis, DMEM medium that contained 100 nM dexamethasone and 50 mg/ml indomethacin (Sigma, USA) was used to induce differentiation in the confluent cell culture for 3 weeks. To induce the cartilage differentiation, a micro-mass culture system was used. For this purpose, 2.5×10^5^ passage-3 cells were pelleted under 1200 g for 5 minutes and cultured in a DMEM medium supplemented with 10 ng/ml transforming growth factor-b3, 10 ng/ml bone morphogenetic protein-6 (BMP-6), 50 mg/ml insulin-transferrin-selenium+premix, 1.25 mg bovine serum albumin and 1% fetal bovine serum (All from Lonza Walkersville Inc, USA).

At the end of osteogenic differentiation, alizarin red (Sigma, USA) staining was used to observe matrix mineralization. After inducing the adipogenesis of stem cells, the cultures were stained by Oil red-O. To induce cartilage differentiation, the micro-mass culture system was used. The prepared sections were then stained by toluidine blue (Sigma, USA).

### Platelet-rich plasma preparation

PRP was prepared using a technique described by Okuda et al. (23). Briefly, 60 mL of autologous blood withdrawn from each dog was initially centrifuged at 2400 rpm for 10 minutes to separate PRP and PPP portions from the red blood cell fraction. The PRP and PPP portions were again centrifuged at 3600 rpm for 15 minutes to separate PRP from PPP. The resulting pellet of platelets was resuspended in 3.0 ml of residual plasma. The PRP was activated at the time of application with a 10% calcium chloride solution (Sigma, USA) and 5000 U of bovine thrombin (Sigma, USA).

### Ilizarov application

The lengthening procedure was performed by applying an Ilizarov system to the left tibia of each animal. All frames were identical and consisted of one proximal and one distal 3/4 ring with a 100.0-mm diameter connected by three treaded rods (6.0 mm diameter) with a 1.0-mm pitch. Under general anesthesia and sterile conditions a distractor was attached to the left tibia using two 1.8-mm diameter trans-osseous wires on both the proximal and distal portion of the tibia. The distractor was placed parallel to the long axis of the tibia and secured by wires. All wires were tensioned with a tensioner. In all dogs, osteotomy of the tibia and fibula was performed in the diaphysis at the level of two thirds of the tibial length fromits distal aspect by use of the multiple drill hole technique. The animals received ketoprofen (2.0 mg/kg/12 hours, intravenous) as an analgesic for three days after surgery. Full loading of the limbs was permitted immediately after surgery. After the operation, the segments were kept in the existing condition for seven days (period of latency) as necessary for the formation of the primary callus and soft tissue healing.

### Distraction process and mesenchymal stem cells transplantation

The bone segments were moved apart at two distraction rates to a maximal length of 30% of the tibia length. During the first session, distraction was performed at a rate of 0.5 mm twice daily (1.0 mm/day). When the lengthening was at half the desired result (15% of the tibia length), BM-derived passage-3 MSCs (1.0×10^7^) and PRP (3.0 ml) were applied in the treatment group to the primary callus. Under fluoroscopy, two 18-gauge needles were inserted at the center of the distracted callus, face-to-face with each tip. We simultaneously injected one ml of a calcium-thrombin mixture and 3.0 ml of MSCs and the PRP suspension into the callus so that the PRP gel might develop within the injected site (17). Similarly, in control group dogs, PRP alone was locally applied to the primary callus. Following the first transplantation, the second session of distraction was carried out at rate of 0.75 mm twice daily (1.5 mm/day) until lengthening was achieved to 30% of the total tibia length. The second transplantation was performed at the end of the distraction period; the distractor was removed and an interlocking nail (7.0 mm) was put in its place.

### Radiography and computerized tomography (CT) scan

Immediately after surgery and twice weekly there after we obtained radiographs of the left tibia. The amount of mineralization of primary callus (immature bone) and mature bone tissue were semi-quantitatively assessed by radiographs. Before bone segments were fixed in formalin, CT scan images were obtained in the longitudinal and transverse aspects of the left intact tibia. The critical outcomes (healing parameters) such as the formation of external (periosteal) callus, intercortical callus, the density of the callus, and the elimination of the gap were semi-quantitatively defined by a radiologist who was blinded to the group assignment of the animals. The scores for each index are tabulated in tables 1-4. CT scan evaluation was performed in the same manner.

**Table 1:** Index of callus density


Description	Score

No callus density	0
Mild callus density	1
Moderate callus density	2
Severe or high callus density	3
Callus density similar to intact peripheral bone	4


**Table 2:** Index of elimination of the gap


Description	Score

Clearly observation of the gap	0
1/4 filled gap	1
1/2 filled gap	2
3/4 filled gap	3
Complete filled gap	4


**Table 3:** Index of formation of the external callus


Description	Score

No callus formation	0
Beginning of callus formation	1
Bridge formation of the external callus with an obvious gap	2
Bridge formation of the external callus with a muffle gap	3
Completed callus and elimination of the gap	4


**Table 4:** Index of formation of intercortical callus


Description	Score

No callus formation	0
Beginning of the callus formation	1
Intercortical formation of callus and its recognition fromthe cortex	2
Intercortical formation of callus and possibility ofcallus recognition from the cortex	3
Completed callus without possibility of callusrecognition from the cortex	4


### Histopathologic analysis

Six months after the initial surgery, the dogs were euthanatized with a barbiturate overdose and the interlocking nail was removed. The left tibias were harvested following intra-arterial vital perfusion of a 10% neutral buffered formaldehyde solution (Sigma, USA). The tibias were routinely fixed and decalcified. The distraction area was sectioned from the central zone to the peripheral zone. In the present study, we investigated the amount of bone in the distraction gaps in four areas that were equally cut from the center of the gap toward the margins with hematoxylin and eosin (H&E). In each area, the sections were obtained in parallel. Each 10 section (8.0 μm) was examined and photographed with an optical microscope (BX60, Olympus, Tokyo, Japan).The extent of new bone formation (distraction-induced bone) on the photomicrographs of the control and treatment sites was quantitatively measured by an examiner using a digital image analyzer system (SigmaScan Pro5, SPSS, Inc., Chicago, IL, USA). For this purpose, the area filled by bone trabeculae in ten randomized microscopic fields was considered. For each dog, mean bone regeneration in the lengthening zone was determined and used for statistical analysis.

### Statistical Analysis

Radiographic, CT scan, and histomorphometric data were compared between control and treatment groups with the Mann-Whitney test. Analysis was performed using commercially available statistical software, the SPSS 15 statistical package (SPSS Inc., Chicago, IL, USA). P<0.05 was considered significant.

## Results

### Mesenchymal stem cells culture and differentiation potential

**Fig.1:**
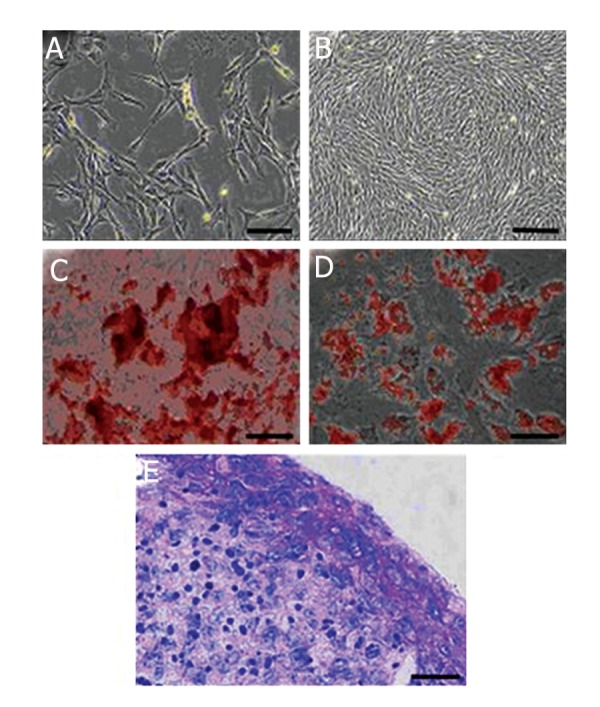
Mesenchymal stem cell (MSCs) culture. A. Photomicrograph of undifferentiated MSCs in primary culture at day 5 (bar=100 μm), B. Photomicrograph of undifferentiated MSCs in confluent passage-3 culture (bar=200 μm), C. Inosteogenic culture, mineralized matrix formed by passage-3 MSCs stained red by the alizarin red staining method (bar=100 μm), D. In adipogenic culture, lipid droplet developed in passage-3 MSCs stained red with the Oil red O staining method (bar=100 μm) and E. In chondrogenic culture, the matrix deposited among passage-3 MSCs stained purple by the toluidine blue staining method (bar=100 μm).

The primary cultures of the canine’s BM mononuclear cells contained some fibroblastic cells as well as a few small, round cells (Fig.1A). The number of small, round cells was reduced by performing subcultures during which the fibroblastic MSCs were purified and expanded. (Fig.1B).

Fibroblastic cells isolated in this study were differentiated into bone, cartilage, and fat cells as confirmed by alizarin red staining for mineralized matrix (Fig.1C), Oil red-O staining for lipid droplets of adipocytes (Fig.1D), and toluidine blue staining for the metachromatic matrix of cartilage (Fig.1E).

### Clinical findings

The mean lengthening of the tibia was 60.8 mm (range: 56 to 66 mm) in both control and treatment groups. The period of lengthening was 50.0 ± 4.0 days (treatment) and 48.2 ± 3.4 (control) days. The period of consolidation was 100.0 ± 8.0 days (treatment) and 96.4 ± 6.8 (control) days and the rate of lengthening was 61.2 ± 3.6 mm (treatment) and 60.4 ± 3.0 mm (control).

### Radiographic findings

**Fig.2:**
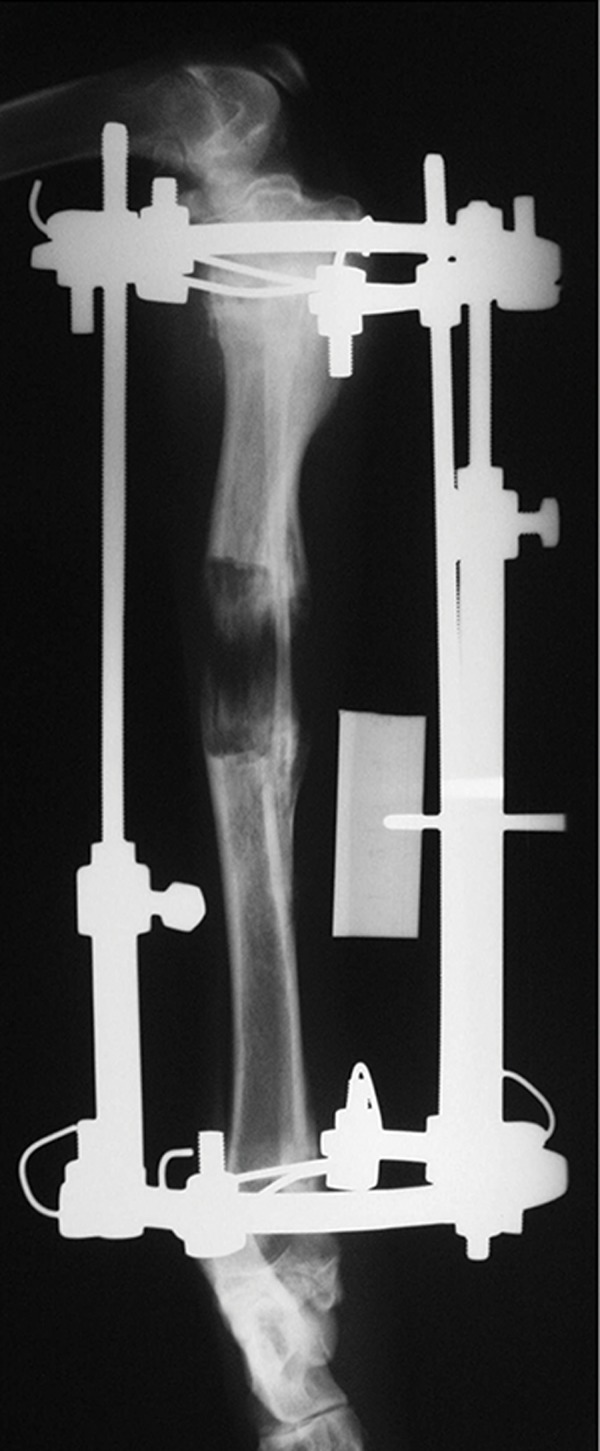
New bone formation was observed in radiograph obtained four weeks after surgery.

Periosteal new bone formation adjacent to the osteotomy site was observed radiographically in both groups as early as two weeks after surgery. The amount and density of periosteal bone increased over time. In the distraction zone, new bone formation was observed four weeks after surgery which at that time periosteal bone had merged with bone (Fig.2). The semi-quantitative evaluation of radiographs, measured by previously described indices revealed a significantly higher amount and quality of callus formation in the treatment group compared to the control group (P<0.05, Table 5).

### Computerized tomography scan findings

On the basis of CT scan image examinations, measurements of the healing parameters (critical outcomes) in dogs of the treatment group were significantly greater than the control group (P<0.05, Table 6).

**Table 5:** Mean ± SE of radiographic healing parameters in the treatment and control groups


Group	Callus density	Elimination of the gap	Formation of external callus	Formation of intercortical callus

Control	1.60± 0.40	1.60± 0.40	2.20± 0.20	1.80 ± 0.37
Treatment	3.00± 0.32^a^	3.00± 0.32^a^	1.40± .40	2.80 ± 0.49


SE; Standard error and ^a^; Significant differences (P<0.05).

**Table 6:** Mean ± SE of CT Scan healing parameters in the treatment and control groups


Group	Callus density	Elimination of the gap	Formation of external callus	Formation of intercortical callus

Control	1.40± 0.24	1.40± 0.40	2.20 ± 0.37	2.00 ± 0.45
Treatment	2.80± 0.37^a^	2.80± 0.20^a^	1.40 ± 0.51	3.40 ± 0.40


SE; Standard error, CT; Computerized tomography and ^a^; Significant differences (P<0.05).

### Histomorphometric findings

**Fig.3:**
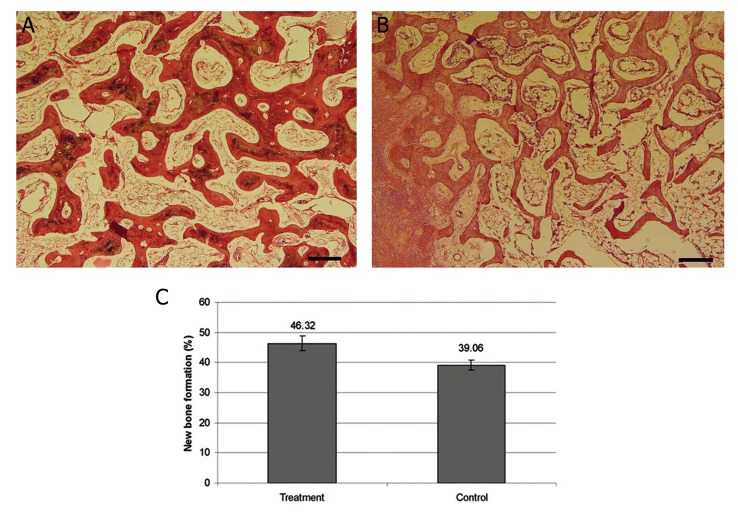
Histomorphometry of the new bone formation in the treatment and control groups. A. Photomicrographs of the peripheral zone fromthe treatment, B. and control group. Width, length, maturation and quality of the formed trabeculae in treatment group are more prominent compared to the control group [hematoxylin and eosin (H&E), bar =100 μm] and C. A graph indicating the relative quantity of newly formed bone in the treatment and control groups. There were significant differences between these groups (P<0.05).

In histological examination, four zones in the area of bone distraction were observed: i. a central zone with collagenous fibers mainly situated parallel to the axis of the lengthened bone. ii. A transitional zone with mineralized osteoid tissue that formed woven bone trabeculae that exhibited low maturation and the presence of active osteoblasts. iii. A remodeling zone with evidence of an intense remodeling process and the presence of osteoclasts. All histological evidence indicated intramembranous bone formation. iv. A peripheral zone composed of mature bone trabeculae that had inserted perpendicular to the central zone of the distraction area and merged with bone tissue in the margin of the gap. The setrabeculae are indicated by lamella-like structures and BM space formation. A mesenchymal soft tissue layer located between trabeculae, especially in the transitional and remodeling zones which included abundant newly formed blood vessels. In all samples, the distractive area was filled with mature connective tissue that was extensively replaced with bone trabeculae and bridged the gap completely. Bone formation was intramembranous and there was no fibrocartilaginous callus remnants or endochondral bone formation at the distraction sites. In the treatment group, the length, maturation, and the quality of the formed trabeculae were more prominent in all four zones when compared to the control group (Fig.3A, B). In some of the treatment cases, a number of lamella-like structures were observed. In the treatment group, histomorphometric evaluation revealed significantly higher mean new bone formation (mean: 46.3%) compared with the control group (mean: 39.1%, P<0.05, Fig.3C). However, in the treatment group the maturity of formed bone, especially in the peripheral zone, was relatively similar to mature natural bone at the undisturbed sites (Fig.3A, B).

## Discussion

Congenital or acquired pathology such as trauma, surgery, and tumors may lead to extensive bone defects and require the transplantation of bone tissue or bone substitutes to restore physiologic structural integrity and function (24). It should be mentioned that one of the most important clinical aspects of limb lengthening and DO is the length of the treatment period; complications during limb lengthening procedures increase in relation to the period of external fixation (25). Although the current gold standard is the use of autologous cancellous bone grafts (24), because of the complications mentioned previously many attempts have been made to develop new methods of treatment (6, 24). In fact, experimental studies are evaluating different ways to accelerate bone healing in different types of animal models. Dog tibial bone is a suitable model for evaluating the effects of different types of treatment on DO in comparison to other models such as rabbit calvarium or rat mandible. In dog tibia, angiogenesis in the bone morrow enhances in response to exercise, resulting in more bone formation and less bone resorption (26). In addition, weight-bearing appears to impact the speed of bone regeneration and maturation (27). Among different strategies used to promote bone regeneration during the lengthening process, it seems that stem cell therapy is one of the most effective ways to improve different aspects of bone healing (25). One possible effective cell therapy is the application of BM-MSCs (24). This study has shown that transplantation of BM-MSCs in combination with PRP positively affected healing during the DO process when compared with PRP alone. Thus, this treatment could shorten the period of lengthening and accelerate the osteogenesis quality along the gap of osteotomized tibia. In primary studies, it has been shown that the transplantation of fresh BM cells can accelerate bone regeneration in DO models (28). However, currently the use of BM-MSCs instead of fresh BM can achieve more acceptable clinical results. MSCs are non-hematopoietic stromal cells that can be used to facilitate fracture healing by accelerating callus formation (29). Following an injury, these cells divide and secrete bioactive factors that stimulate angiogenesis and vasculogenesis, and are mitotic to tissue-intrinsic progenitor cells (30). Experiments have revealed the efficacy of BM-MSCs for enhancing bone regeneration and mineralization. In addition, experiments have proven that MSCs loaded onto scaffolds can heal large defects, and culture-expanded MSCs bridge critical size defects in animal models (31). Although other sources of stem cells, such as osteoblast- like cells derived from the periosteum can be used for this purpose (32), the use of BM-MSCs is more clinically acceptable because they are relatively easy to isolate, can be expanded rapidly in vitro and have the capacity to be differentiated into multiple cell types *in vivo*. In addition, their application seems to be safe and without complications or ethical concerns (29). Application of BM-MSCs is reported to be related to higher amount of newly formed bone compared to the adipose tissue derived stem cells in critical size defect of the sheep tibia (33). The selection of a suitable carrier for cell transplantation in bone healing is important to achieve the best results. It has been demonstrated that critically sized defects in dog long bone scan be healed by the use of culture-expanded autologous osteogenic stem cells combined with porous ceramic implants (31). However, it is important to mention that BM-MSCs loaded on ceramics will not be able to form a natural bone tissue due to the slow degradation of the scaffold material (31). Among different types of carriers and scaffolds for this purpose, it appears that PRP has many benefits. Many experimental studies demonstrate positive effects of using PRP as a carrier of MSCs for transplantation in bone defects. PRP gel has been used successfully as a scaffold for bone formation (34) and it enhances bone regeneration when used in conjunction with autologous bone graft in the field of reconstructive oral and maxillofacial surgery (35). It has been shown that PRP in combination with autologous cancellous graft leads to a significantly better bone regeneration compared to isolated application of autologous cancellous graft in an *in vivo* critical size defect on load-bearing long bones of mini-pigs (36). PRP osteoconductive fibrin clots have several signaling molecules, such as platelet-derived growth factor (PDGF) (29), which are necessary for MSC survival and proliferation (37). Other growth factors present in PRP include BMPs, insulin-like growth factor (IGF) and fibroblast growth factor (FGF), all of which seem to have numerous positive effects that include mitogenesis, angiogenesis, and the upregulation of other growth factors (19, 37).

Additionally, these growth factors inhibit osteoclast formation and bone resorption and possibly increase the number of osteoblasts, thereby accelerating bone formation (38). On the whole, the addition of MSC in PRP scaffold has increased new bone formation, mineralization, and mechanical property compared to the PRP-only group (21). In the present study, we used autologous fresh PRP. The rate of distraction in the DO process had an important role in achieving the best results. From a mechanobiological point of view, a poor osteotomy, frame instability, and a high distraction rate might all disturb vascularization and local blood supply to the regenerating tissue, thereby causing delayed bone heading (39). It has been shown that a rapid distraction rate causes the disruption of blood vessels in the distraction zone (40). Ilizarov determined that onemm per day (0.25-mm lengthening, four times a day) resulted in acceptable new bone formation (1). In dogs, a distraction rate of 0.5 mm per day has been shown to result in premature consolidation after approximately 10 days (1). It should be mentioned that this rate could be changed depending on the status of the callus and tension within the soft tissues (25). These standards have been used in the present study. On the whole, we can conclude that autologous cell therapy for bone regeneration by a combination of MSCs and PRP has many advantages for clinical feasibility. These findings may be applicable in the repair of bone defects and can be a useful alternative to allogenic or autologous bone grafts because the proposed treatment is safe, minimally invasive, easy to perform, and has great potential for clinical applications.

## Conclusion

Taken together, the transplantation of BM-MSCs positively affects early bony consolidation in DO. The use of MSCs along with PRP may allow a shortened period of consolidation and therefore permit earlier device removal.
